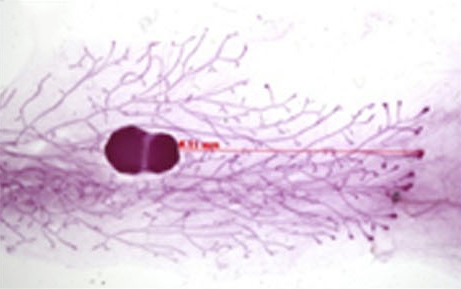# RUNX2: a prognostic biomarker for triple-negative breast cancer?

**Published:** 2014-05

Abnormal expression of the RUNX family of transcription factors, members of which regulate developmental cell specification during haematopoiesis and osteogenesis, can promote or suppress tumour formation in a context-specific manner. Although RUNX2 has a known oncogenic role in some cancer types and is upregulated in metastatic breast cancer cell lines, its role in primary epithelial cancers is poorly understood. To investigate the role of RUNX2 in one of the most common forms of epithelial cancer, Karen Blyth and colleagues analysed *RUNX2* expression in human breast cancer tumours. Tissue microarray analysis of tumour samples revealed that high *RUNX2* expression correlates with the triple-negative breast cancer subtype and with lower patient survival rates compared with samples displaying low *RUNX2* expression. To explore the effects of *RUNX2* overexpression in normal mammary tissue, the authors generated a new transgenic mouse model. They report that overexpression of *Runx2* disrupts normal mammary development in young mice and potentiates pre-neoplastic lesions in aged mice. Together, these results indicate that *RUNX2* expression is tightly regulated and might function in the maturation of normal breast epithelia. Importantly, the study tentatively suggests that RUNX2 could serve as a prognostic biomarker for triple-negative breast cancer and provides a foundation for elucidating the molecular events involved in this aggressive cancer subtype. **Page 525**

This summary was written by Anna J. Moyer, who is currently in the third year of a dual Bachelor’s/Master’s programme in Biology at the University of Alabama.

**Figure:**